# Bridging Three Decades: Global Self-Harm Trends From 1990–2021 and Projections to 2040

**DOI:** 10.62641/aep.v54i3.2111

**Published:** 2026-06-15

**Authors:** Hong Zhu, Xuhong Zhang, Tiantian Chen, Zhixin Lim, Saboor Saeed, Jianping Tong, Ye Shen

**Affiliations:** ^1^Department of Ophthalmology, The First Affiliated Hospital of Zhejiang University, 310003 Hangzhou, Zhejiang, China; ^2^Cixi Mingguang Eye Hospital, 315300 Ningbo, Zhejiang, China; ^3^School of Medicine, Zhejiang University, 310003 Hangzhou, Zhejiang, China; ^4^Nanhu Brain Computer Interface Institute, 310003 Hangzhou, Zhejiang, China

**Keywords:** self-injurious behaviour, global health, epidemiology, health inequities, forecasting

## Abstract

**Background:**

Self-harm, which includes both non-suicidal self-injury and suicidal behaviors, poses a major global public health challenge. This study provides a comprehensive analysis of trends in self-harm worldwide, the socioeconomic disparities associated with it, and future projections, using data from the Global Burden of Disease, Injuries, and Risk Factors Study (GBD) 2021.

**Methods:**

Self-harm data were extracted from GBD 2021, including incidence, prevalence, mortality, years of life lost (YLLs), years lived with disability (YLDs), and disability-adjusted life years (DALYs) for 204 countries and territories from 1990 to 2021. Age-standardized rates and estimated annual percentage change (EAPC) were calculated. Inequality was assessed using the Slope Index of Inequality (SII) and Concentration Index (CI). Autoregressive Integrated Moving Average (ARIMA) models were employed to generate projections of self-harm burden from 2022 to 2040.

**Results:**

The global burden of self-harm is projected to change substantially by 2040, with deaths estimated to increase to 829,853 (95% Uncertainty Interval (UI), 262,233–1,397,474) and prevalence projected to rise to 35,863,341 (95% UI, 8,079,108–63,647,574) cases (representing a 131.9% increase from the 2021 baseline of 15,467,153 cases). From 1990 to 2021, age-standardized rates of self-harm demonstrated decreasing trends globally and across sociodemographic index (SDI) levels, with the largest declines observed in high-middle SDI countries. Gender disparities were evident, with more pronounced decreases in females. Inequalities in DALYs due to self-harm decreased over time but remained higher among females in lower-SDI populations.

**Conclusions:**

Despite decreasing age-standardized rates, the global burden of self-harm is projected to increase substantially by 2040, with driven by increasing incidence and prevalence in incidence and prevalence. Inequities persist, particularly among females in lower-SDI populations. Implementation of targeted prevention and intervention strategies, strengthening of mental health systems, and addressing social determinants of health are imperative to reduce the growing burden of self-harm worldwide.

## Introduction

Self-harm, which includes both non-suicidal self-injury and suicidal behaviour, represents a pressing health concern globally, leading to considerable morbidity, mortality, and socioeconomic costs [1, 2]. Despite heightened awareness and prevention efforts, self-harm continues to be a pervasive issue worldwide [3], exhibiting different trends across various regions and demographic groups [4]. Approximately 700,000 people die by suicide each year, representing a significant public health burden, with self-harm episodes being substantially more frequent than suicide deaths [5].

The Global Burden of Diseases, Injuries, and Risk Factors Study (GBD) [6] has become a vital resource for understanding the global epidemiology of self-harm. Previous versions of the GBD have highlighted the complex factors contributing to the burden of self-harm, including socioeconomic conditions, cultural norms, access to mental health services, and the prevalence of mental disorders [7, 8]. The GBD 2021 represents a significant advancement over prior iterations, incorporating updated data sources from vital registration systems and verbal autopsy studies, refined methodological adjustments for under-reporting and misclassification of self-harm deaths, and expanded geographic coverage across 204 countries and territories [9]. Additionally, GBD 2021 includes data through 2021, capturing the potential impact of the COVID-19 pandemic on self-harm trends, which was not available in earlier versions such as GBD 2019.

While previous studies have explored self-harm trends in specific countries or regions [10, 11], there is currently a lack of comprehensive analysis addressing global trends, socioeconomic disparities, and future projections. Such analysis is essential for informing policy decisions, allocating resources, and creating targeted interventions to reduce the global burden of self-harm [12].

Recent research emphasizes the importance of assessing both fatal and non-fatal self-harm in evaluations of health burden. Non-fatal episodes play a significant role in the overall health burden and are strong predictors of future suicidal behavior [13, 14]. Furthermore, the COVID-19 pandemic has likely intensified mental health challenges worldwide, underscoring the necessity for current analyses of self-harm trends [15]. Some studies have provided emerging evidence on pandemic-related impacts. A study by Hawton *et al*. [16] reported increased rates of self-harm presentations among adolescents during lockdown periods in high-income countries, while Pirkis *et al*. [17] found that national suicide rates in 21 countries remained relatively stable in the early pandemic months, potentially reflecting rapid implementation of crisis services and social protection measures. Importantly, our dataset extends through 2021, capturing the initial years of the pandemic. However, disentangling the specific impact of the pandemic from underlying long-term trends requires careful interpretation, as the full effects of COVID-19 on self-harm patterns may not yet be fully reflected in available data.

This study provides a comprehensive analytical framework integrating the following elements: (1) extended temporal coverage spanning 31 years (1990–2021) using the most recent GBD 2021 data; (2) simultaneous analysis of six key epidemiological indicators (incidence, prevalence, mortality, years of life lost (YLLs), years lived with disability (YLDs), and disability-adjusted life years (DALYs)) capturing both fatal and non-fatal self-harm burden; (3) systematic quantification of socioeconomic inequalities using validated metrics including the Slope Index of Inequality (SII) and Concentration Index (CI); (4) Autoregressive Integrated Moving Average (ARIMA)-based projections extending to 2040 to inform long-term policy planning; and (5) granular analysis across 204 countries and territories stratified by SDI quintiles and sex.

This study aims to address these gaps by leveraging the most recent data from the GBD 2021 study to provide a comprehensive analysis of global self-harm trends from 1990 to 2021. We examine incidence, prevalence, mortality, YLLs, YLDs, and DALYs associated with self-harm. Additionally, we investigate socioeconomic inequalities in the burden of self-harm and project future trends through 2040.

Through this analysis, we aim to offer valuable insights to researchers, clinicians, and policymakers involved in preventing self-harm and alleviating its effects on individuals and society. Gaining an understanding of the changing landscape of self-harm is essential for developing effective prevention strategies, optimizing resource allocation, and ultimately diminishing the global impact of this significant public health issue [18].

## Methods

### Data Source

This study utilized data on self-harm from GBD 2021, covering the period from 1990 to 2021. Data were extracted from the Global Health Data Exchange (GHDx) on October 15, 2024, using the GBD 2021 final release (GBD Results Tool, available at https://vizhub.healthdata.org/gbd-results/).The GBD 2021 provides comprehensive estimates more than 350 diseases and injuries across 204 countries and territories .We specifically extracted data related to self-harm, including incidence, prevalence, mortality, YLLs, YLDs, and DALYs. The data were stratified by year, age group, sex, and geographic location [19].

Self-harm in the GBD 2021 is defined as intentional self-inflicted poisoning or injury, which may or may not have fatal intent or outcome. This broad definition aligns with recommendations from the WHO and the International Association for Suicide Prevention (IASP), which recognize that distinguishing intent in self-harm is often unreliable, particularly in retrospective data collection and across diverse cultural contexts [20]. It is important to note that the GBD framework does not distinguish between NSSI and suicidal behaviors (e.g., suicide attempts, completed suicide), as both are captured under the broader category of self-harm. This inclusive definition reflects the challenges in reliably ascertaining intent across diverse global settings, where data sources vary in their ability to document suicidal intent. While this approach ensures consistency and comparability across countries and time periods, readers should be aware that our findings encompass a heterogeneous spectrum of self-harm behaviors with potentially different etiologies and clinical implications. The recent Lancet Commission on self-harm (2024) has similarly adopted this inclusive approach, acknowledging that NSSI and suicidal behaviors often co-occur and share common risk factors, while also recognizing their distinct characteristics [21]. This definition encompasses a range of self-harming behaviors, from non-suicidal self-injury to suicide attempts and completed suicides.

### Burden Description

Age-standardized incidence rate (ASIR), age-standardized prevalence rate (ASPR), age-standardized mortality rate (ASMR), and age-standardized DALY rate (ASR DALY) were used to study the global patterns and trends of self-harm burden [4]. The relationship between SDI and these metrics across 21 GBD regions and 204 countries was analyzed to illustrate the inequalities in self-harm burden related to socioeconomic development [22].

### Cross-Country Inequality Analysis

The distributional inequality in the burden of self-harm across countries was assessed using the SII and the CI [23]. The SII measures the absolute difference in health outcomes between the most and least advantaged groups, whereas the CI quantifies the relative concentration of health outcomes across the socioeconomic spectrum [24].

### Trends Analysis

Trends in the burden and inequality associated with self-harm were analysed using the estimated annual percentage changes (EAPC) [25]. The relative change in SII and CI between 1990 and 2021 was calculated.

### Time Series Forecasting

ARIMA models were used for time series forecasting to predict trends from 2022 to 2040 for each measure and gender category [26]. ARIMA was selected for its suitability in epidemiological time series forecasting, as it effectively handles non-stationary data commonly observed in disease burden trends through differencing, and appropriately accounts for autocorrelation structures inherent in historical epidemiological data. Furthermore, ARIMA models have demonstrated robust performance in previous GBD-related forecasting studies, supporting their application in the present analysis.

The model parameters (p, d, q) were selected using the Hyndman-Khandakar algorithm implemented in the auto.arima function in R, which systematically evaluates combinations of autoregressive order (*p* = 0–5), differencing order (d = 0–2), and moving average order (q = 0–5), selecting the specification that minimizes the corrected Akaike Information Criterion (AICc) while ensuring model parsimony. For each outcome measure (incidence, prevalence, mortality, and DALYs), separate ARIMA models were independently calibrated for the overall population, males, and females, allowing the algorithm to capture sex-specific temporal patterns and dynamics.

### Data Availability and Ethics

The data for this study are publicly available on the GHDx website (http://ghdx.healthdata.org/), which is accessible to the public. The study adhered to the Guidelines for Accurate and Transparent Health Estimates Reporting (GATHER) statement [27].

### Statistics

Statistical analyses were performed using R (version 4.3.3; R Foundation for Statistical Computing, Vienna, Austria). and Stata (version 17.0; StataCorp LLC, College Station, TX, USA). In R, the following packages were utilized: forecast (version 8.21) for ARIMA modeling and time-series projections via the auto.arima function, ggplot2 (version 3.4.4) for data visualization, dplyr (version 1.1.4) for data manipulation, and tidyverse (version 2.0.0) for general data processing. In Stata, the conindex module was used to calculate concentration indices, and custom commands were employed to compute the SII based on weighted least squares regression. Age-standardized incidence rates (ASIR), age-standardized prevalence rates (ASPR), age-standardized mortality rates (ASMR), and age-standardized DALY rates were calculated per 100,000 population with 95% uncertainty intervals (UIs) [13]. Health inequalities were assessed using the SII and CI, calculated with 95% confidence interval (CI) derived from 1000 bootstrap replications. Trends were analysed using EAPC, calculated with 95% CIs using Joinpoint regression. Countries were categorized into sociodemographic index (SDI) quintiles for subgroup analyses. Based on GBD 2021 classifications, the SDI quintiles were defined as follows: low SDI (SDI <0.47), low-middle SDI (0.47 ≤ SDI < 0.62), middle SDI (0.62 ≤ SDI < 0.71), high-middle SDI (0.71 ≤ SDI < 0.81), and high SDI (SDI ≥0.81) [28].

The SDI is a composite measure developed by the Institute for Health Metrics and Evaluation (IHME) that combines lag-distributed income per capita, average years of schooling among individuals aged 15 years and older, and total fertility rate among women younger than 25 years. The index is rescaled from 0 to 1, with higher values indicating greater socioeconomic development.

Representative countries for each quintile include: low SDI (e.g., Chad, Central African Republic, Niger, and South Sudan); low-middle SDI (e.g., India, Pakistan, Kenya, and Bangladesh); middle SDI (e.g., Egypt, the Philippines, South Africa, and Indonesia); high-middle SDI (e.g., China, Brazil, Mexico, and Thailand); and high SDI (e.g., the United States, Germany, Japan, and Australia). These classifications enable readers to contextualize trends within specific developmental settings and facilitate replication of the analysis.

### Model Diagnostics and Evaluation

Residual analysis was performed to assess model adequacy. Specifically, autocorrelation function (ACF) and partial autocorrelation function (PACF) plots were examined to confirm that residuals approximated white noise, indicating no remaining systematic patterns. The Ljung-Box test was applied to verify the absence of significant residual autocorrelation at multiple lag orders. Models that failed these diagnostic checks were re-specified until adequate fit was achieved. All final models passed residual diagnostics, supporting the validity of the forecasting results. Model performance was evaluated using multiple accuracy metrics, including Mean Absolute Percentage Error (MAPE), Root Mean Square Error (RMSE), and Mean Absolute Scaled Error (MASE) [29].

The prediction analysis was conducted using R version 4.3.3, employing a comprehensive statistical approach to forecast self-harm trends. Time series modelling was implemented using the “forecast” package, with visualization performed through “ggplot2”.

Model adequacy was assessed through rigorous residual analysis. The prediction accuracy was evaluated using three key metrics:

∙ Mean Absolute Percentage Error (MAPE):

MAPE = $\frac{1}{n}\,\sum_{t=1}^{n}\left|\frac{A_{t}-F_{t}}{A_{t}}\right|\times 100\%$

∙ Root Mean Square Error (RMSE):

RMSE = $\sqrt{\frac{1}{n}\,\sum_{t=1}^{n}{\left(A_{t}-F_{t}\right)}^{2}}$

∙ Mean Absolute Scaled Error (MASE):

MASE = $\frac{\frac{1}{n}\,\sum_{t=1}^{n}\left|A_{t}-F_{t}\right|}{\frac{1}{n-1}\,\sum_{t=2}^{n}\left|A_{t}-A_{t-1}\right|}$

The model demonstrated robust performance with a MAPE of 1.06%, indicating high prediction accuracy. According to established forecasting benchmarks, a MAPE below 10% is generally considered indicative of highly accurate forecasting, while values below 5% reflect excellent model performance, Previous time series studies of disease burden and mental health outcomes have reported MAPEs typically ranging from 3% to 15%, depending on the outcome complexity and geographic scope. Our MAPE values of approximately 1–2% thus represent exceptionally strong predictive accuracy, comparing favourably with published benchmarks in epidemiological forecasting. The RMSE of 47,477.03 and MASE of 1.28 further confirmed the model’s reliability for forecasting purposes. A MASE value close to 1 indicates that the model performs comparably to a naïve forecast, while values below 1 suggest superior performance; our MASE of 1.28, while slightly above 1, remains within acceptable ranges for long-term epidemiological projections with inherent uncertainty.

Forecasts were generated and visualized using the ggplot2 package in R, with separate visualizations for incidence, prevalence, mortality, and DALYs, stratified by gender. Each visualization incorporates:

∙ Historical data (1990–2021).

∙ Point forecasts (2022–2040).

∙ 95% prediction intervals, represented as shaded regions around the forecast line.

The prediction intervals account for both model uncertainty and inherent variability in the time series, providing a comprehensive view of potential future trajectories.

## Results

### Trends in Age-Standardized Rates of Self-Harm From 1990 to 2021

Globally, age-standardized rates of self-harm showed a consistent declining trend from 1990 to 2021 (**Supplementary Fig. 1**). The age-standardized death rate decreased significantly with an EAPC of –1.97 (95% CI: –2.11 to –1.83), with more pronounced reductions observed in females (EAPC = –2.62, 95% CI: –2.76 to –2.49) compared to males (EAPC = –1.66, 95% CI: –1.81 to –1.50).

When stratified by socio-demographic index (SDI), distinct patterns emerged across different regions. High-middle SDI regions demonstrated the most substantial decrease, particularly in DALYs, showing a marked decline from approximately 800 per 100,000 in 1990 to 350 per 100,000 by 2021. High SDI regions maintained relatively stable rates, with a modest decline in all measures. Low SDI regions consistently showed the lowest absolute rates but maintained a steady downward trajectory.

The burden of self-harm exhibited notable gender disparities across all SDI quintiles. Males consistently showed higher age-standardized rates across all metrics, particularly in DALYs and mortality. The male-to-female ratio was most pronounced in high SDI regions, where male rates were approximately twice those of females. Age-stratified analyses showed distinct sex patterns across the life course. Females had higher non-fatal self-harm rates in adolescence and young adulthood, while males had markedly higher suicide mortality in middle and older age, with male-to-female ratios exceeding 2.5 in midlife and 3.0 in older adults in many high-SDI settings. These interactions indicate the need for age- and sex-specific prevention strategies, emphasizing early intervention for young females and mortality-focused approaches for middle-aged and older males.

**Fig. 1.**
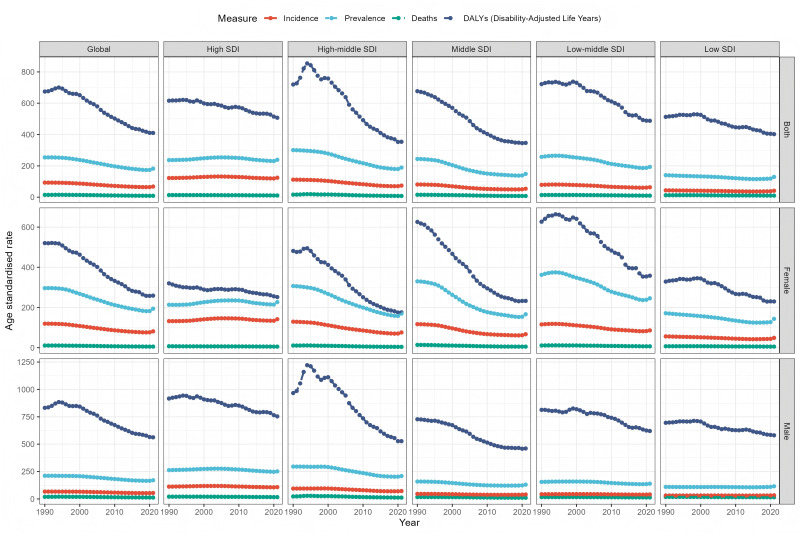
**Global trends in age-standardized rates of self-harm by SDI regions and sex from 1990 to 2021**. The trends are shown separately for both sexes combined (top row), females (middle row), and males (bottom row) across different SDI regions (Global, High SDI, High-middle SDI, Middle SDI, Low-middle SDI, and Low SDI). Four measures are displayed: incidence (red), prevalence (cyan), deaths (green), and DALYs (navy blue). DALYs generally showed the highest rates and most pronounced declining trends across all SDI regions, particularly in high-middle SDI areas. Age-standardized rates per 100,000 population are shown on the y-axis, and years from 1990 to 2021 are shown on the x-axis. Abbreviations: SDI, Socio-demographic Index; DALYs, disability-adjusted life years. Data source: Global Burden of Disease Study 2021.

For incidence and prevalence, the trends showed more modest declines compared to mortality and DALYs, suggesting improvements in case fatality rates over the study period. The prevalence rates demonstrated the smallest relative change among all metrics, particularly in high SDI regions where they remained relatively stable throughout the study period (Fig. 1, **Supplementary Table 1**).

### Decomposition of DALYs Into YLLs and YLDs

Decomposition of disability-adjusted life years showed that YLLs due to premature death accounted for the majority of the global self-harm burden, contributing approximately 85 to 90 percent across the study period. This pattern remained largely stable from 1990 to 2021, with only modest regional variation. In high Socio-Demographic Index regions, the proportion attributable to YLDS increased over time, likely reflecting improved survival and greater recognition of non-fatal consequences. In contrast, low Socio-Demographic Index regions remained dominated by premature mortality, indicating persistently high fatality and probable under-ascertainment of non-fatal self-harm. These findings highlight that while reducing suicide deaths remains the central priority, increasing attention to non-fatal outcomes is warranted, particularly in settings with higher survival.

### Socioeconomic Inequalities in Self-Harm DALYs

**Fig. 2.**
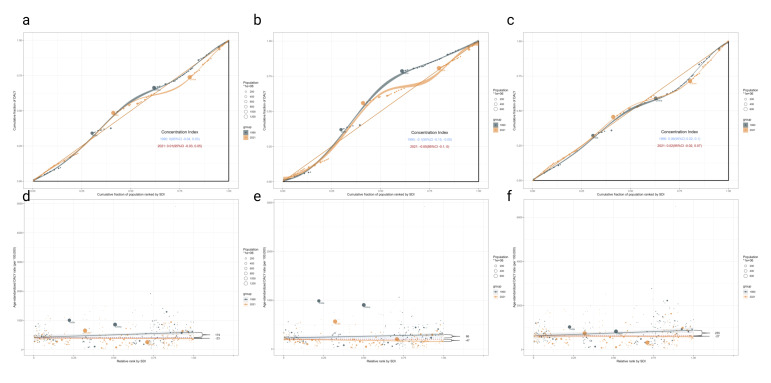
**Trends in socioeconomic inequalities in self-harm DALYs from 1990 to 2021**. (a) Concentration index trends for females, showing changes in relative inequalities. (b) Concentration index trends for males, showing changes in relative inequalities. (c) Overall concentration index trends for both sexes combined. (d) Slope index of inequality trends for females, showing changes in absolute inequalities. (e) Slope index of inequality trends for males, showing changes in absolute inequalities. (f) Overall slope index of inequality trends for both sexes combined. Each point represents a country, with size proportional to population (in millions). China and India are highlighted with labels due to their substantial global population share. The x-axis shows cumulative fraction of population ranked by SDI (a–c) or relative rank by SDI (d–f). Key country values: China CI changed from 0.08 to 0.03; India CI changed from 0.05 to 0.04. Concentration indices range from –1 to 1, with negative values indicating concentration among lower socioeconomic groups and positive values indicating concentration among higher socioeconomic groups. The slope index of inequality represents absolute differences in DALYs per 100,000 population between the highest and lowest socioeconomic positions. The x-axis represents the relative rank by SDI, where countries are ordered from lowest SDI (rank = 0, left) to highest SDI (rank = 1, right), illustrating the distribution of self-harm burden across the socioeconomic spectrum. Abbreviations: DALYs, disability-adjusted life years; SDI, Socio-demographic Index; SII, Slope Index of Inequality; CI, Concentration Index.

Our analysis of concentration indices revealed distinct patterns of socioeconomic-related health inequalities across different demographic groups between 1990 and 2021. The female population showed a notable improvement in health equity, with the concentration index shifting from –0.10 (95% CI: –0.15 to –0.05) in 1990 to –0.05 (95% CI: –0.10 to 0.00) by 2021, indicating a reduction in health disadvantages among lower socioeconomic groups. In contrast, males exhibited a different trajectory, with the concentration index moving from 0.06 (95% CI: 0.02 to 0.10) to 0.02 (95% CI: –0.02 to 0.07) over the same period, suggesting a modest decrease in health advantages among higher socioeconomic groups. The overall population concentration index changed from –0.004 (95% CI: –0.04 to 0.04) in 1990 to 0.006 (95% CI: –0.03 to 0.05) in 2021 (Fig. 2a–c, **Supplementary Table 2**).

The slope indices of inequality (SII) further revealed substantial changes in absolute health inequalities during this period (**Supplementary Table 3**). Among females, the SII decreased from 66.2 (95% CI: –1.4 to 133.7) to –47.5 (95% CI: –92.9, –2.1) DALYs per 100,000 population, indicating a reversal in health inequality patterns. For males, an even more pronounced reduction was observed, with the SII declining from 255.3 (95% CI: 56.8 to 453.9) to –27.0 (95% CI: –184.8, 130.8) DALYs per 100,000 population. The overall population showed a consistent pattern of improvement, with the SII decreasing from 173.9 (95% CI: 46.0 to 301.9) to –23.0 (95% CI: –120.2, 74.1) DALYs per 100,000 population. Within this global context, China demonstrated rapid progress in reducing the burden of self-harm, as evidenced by the substantial decrease in DALY rates (EAPC: –3.77 for males, –5.61 for females), while India showed a more gradual reduction (EAPC: –1.07 for males, –2.27 for females) (Fig. 2d–f, **Supplementary Tables 3 & 4**).

### Relationship Between Key Indicators of Self-Harm and SDI

**Fig. 3.**
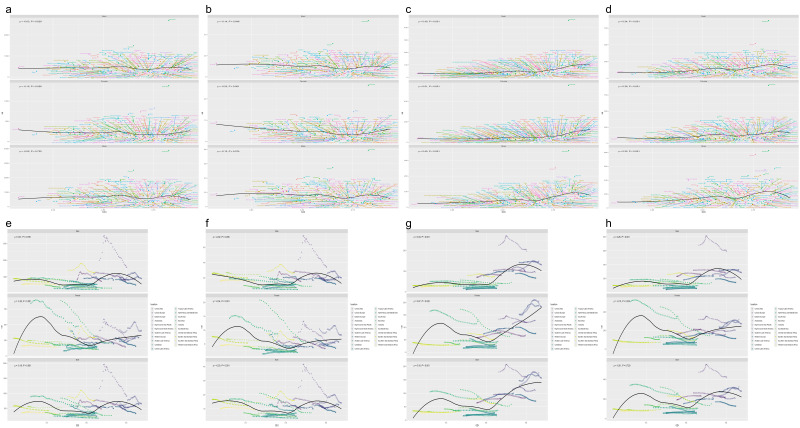
**Age-standardised rates of self-harm by Socio-demographic Index (SDI)**. (a–d) Country-level analysis showing the relationship between SDI and mental health metrics, with countries coloured by region. (a) DALYs per 100,000 population showing varying burden across SDI levels. (b) Death rates per 100,000 population demonstrating inverse relationship with development. (c) Incidence rates per 100,000 population indicating higher rates in developed nations. (d) Prevalence per 100,000 population showing positive correlation with SDI. (e–h) Regional patterns of age-standardised rates by SDI from 1990 to 2021. (e) DALYs per 100,000 population showing distinct regional variations. (f) Death rates per 100,000 population demonstrating regional disparities. (g) Incidence rates per 100,000 population showing regional patterns of new cases. (h) Prevalence per 100,000 population illustrating sustained burden across regions. Each line represents the average relationship between the respective metric and SDI for that region, with Eastern Europe showing notably different patterns. In panels (a–d), each point represents a country, with colours indicating geographical regions. In panels (e–h), each line represents the regional average trend of the respective metric across SDI levels over time. Solid lines indicate modelled trends, and no additional symbols are used. Abbreviations: SDI, Socio-demographic Index; DALYs, disability-adjusted life years.

The relationship between key indicators of self-harm and SDI shows varied trends (Fig. 3a–h, **Supplementary Tables 4, 5**). DALYs and prevalence rates demonstrated weak to moderate positive correlations with SDI, indicating an increase in the overall burden and prevalence of self-harm as social development levels rise. Death rates showed a weak to moderate negative correlation with SDI, particularly for females, suggesting that as social development increases, self-harm death rates tend to decrease. Incidence rates exhibited a moderate positive correlation with SDI, indicating that new cases of self-harm are more common in areas with higher social development. All indicators displayed significant inter-country variations, even at similar SDI levels, highlighting the complexity of self-harm behaviour and the influence of factors beyond SDI.

### Global Burden of Self-Harm: Projections for 2040

**Fig. 4.**
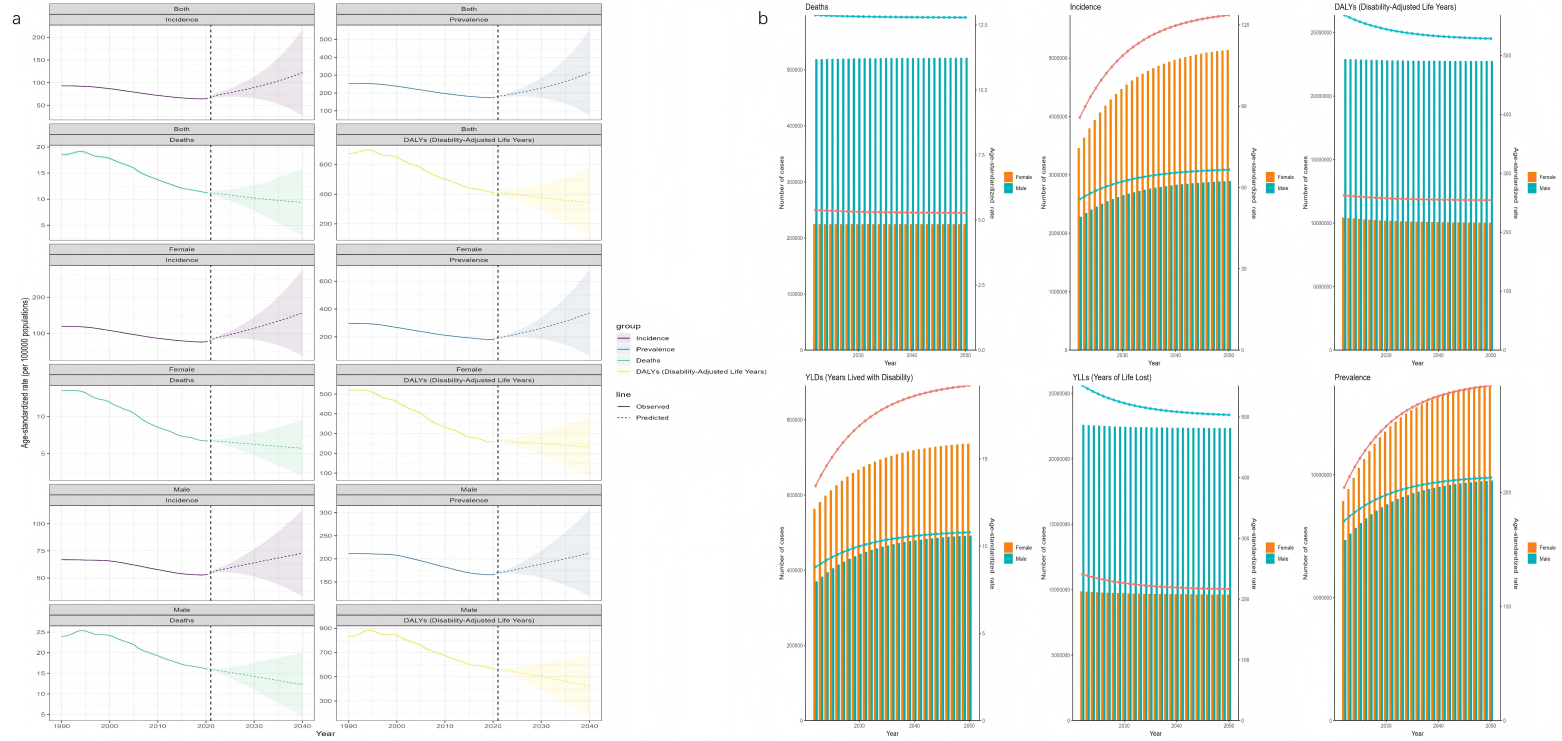
**Observed (1990–2021) and projected (2022–2040) trends in self-harm burden**. Panel (a) shows age-standardized rates per 100,000 population for incidence, prevalence, deaths, and DALYs with 95% uncertainty intervals (shaded areas). Panel (b) shows absolute numbers stratified by sex. Solid lines indicate observed data; dashed lines indicate projections. Wide uncertainty intervals for 2040 projections reflect the extended forecast horizon, data quality heterogeneity, and inherent unpredictability of future events. Central projections indicate increasing absolute burden for deaths, incidence, and prevalence, with DALYs showing potential slight decrease. Abbreviations: DALYs, disability-adjusted life years; UI, uncertainty interval. Key 2040 projections (both gender): deaths 829,853 (95% UI: 262,233–1,397,474); prevalence 35,863,341 (95% UI: 8,079,108–63,647,574). Abbreviations: DALYs, disability-adjusted life years; UI, uncertainty interval. Projection method: Autoregressive Integrated Moving Average (ARIMA) models.Note: Panel (a) displays age-standardized rates per 100,000 population, which show declining trends for most indicators. Panel (b) displays absolute numbers, which show increasing trends due to population growth and demographic transitions. This divergence between rate-based and count-based measures has important implications for health system capacity planning.

The global burden of self-harm is projected to change substantially by 2040 (Fig. 4 and **Supplementary Fig. 2**). For both sexes combined, deaths are estimated to increase to 829,853 (95% UI, 262,233–1,397,474) in 2040 from 746,277 (95% UI, 733,173–759,380) in 2021. DALYs are projected to decrease slightly to 34,126,562 (95% UI, 11,854,769–56,398,355) in 2040 from 33,524,973 (95% UI, 33,440,859–33,609,087) in 2021. Incidence is expected to rise to 11,757,944 (95% UI, 2,626,264–20,889,625) cases in 2040 from 5,487,418 (95% UI, 5,453,325–5,521,513) cases in 2021. Prevalence is projected to increase markedly to 35,863,341 (95% UI, 8,079,108–63,647,574) cases in 2040 from 15,467,153 (95% UI, 15,407,100–15,527,206) cases in 2021. Gender differences are evident in the projections, with males having higher estimated deaths at 561,815 (95% UI, 184,946–938,684) and DALYs at 22,329,286 (95% UI, 8,212,896–36,445,676) in 2040 compared to females with deaths projected at 268,038 (95% UI, 77,287–458,790) and DALYs at 11,797,276 (95% UI, 3,641,873–19,952,679) in 2040. While the absolute number of cases is projected to increase due to population growth, the age-standardized incidence rates are projected to decline (Fig. 4a).

These projections suggest a concerning upward trend in self-harm burden, particularly in terms of incidence and prevalence, despite a potential slight decrease in overall DALYs (Fig. 4, **Supplementary Tables 6, 7**). The wide UIs indicate substantial variability in these long-term projections.

The projections indicate a continued increase in self-harm incidence and prevalence, despite a modest decline in overall DALYs, with wide UIs reflecting substantial long-term variability. This uncertainty arises from the extended forecast horizon, regional heterogeneity in data quality, particularly in low-SDI settings, potential distortions from pandemic-era data, and the inability of trend-based models to account for future policy, socioeconomic, or external shocks. While point estimates should be interpreted cautiously, the consistent direction of trends across uncertainty ranges supports the conclusion of a rising absolute self-harm burden.

## Discussion

This comprehensive analysis of the global burden of self-harm, utilizing data from the Global Burden of Diseases, Injuries, and Risk Factors Study (GBD) 2021, provides valuable insights into the evolving landscape of this critical public health issue. The findings reveal the complex interplay between socioeconomic development, gender disparities, and the changing epidemiology of self-harm worldwide.

### The Uniqueness of the Current Study: A Comprehensive Approach to Self-Harm Epidemiology

This study presents a comprehensive analysis of the global burden of self-harm using the most recent GBD 2021 dataset, integrating long-term trends, inequality measures, and future projections. Unlike previous studies that concentrated on specific aspects of self-harm epidemiology, such as suicide mortality trends [4] or the prevalence of suicidal thoughts and behaviors in particular populations [11], the current study offers a more comprehensive perspective. It examines a broad range of self-harm-related metrics including incidence, prevalence, mortality, YLLs, YLDs, and DALYs across 204 countries and territories, thereby providing a more holistic view of the global burden of self-harm. Additionally, the study’s analysis of socioeconomic disparities and long-term projections distinguishes it from previous research, yielding valuable insights into the future trajectory of self-harm and the factors that may influence it [30, 31]. This comprehensive approach fosters a deeper understanding of the complex nature of self-harm and underscores the importance of multifaceted prevention and intervention strategies. Unlike previous studies, the present study provides a more integrated perspective by combining three decades of trend analysis with projections to 2040 and a systematic evaluation of socioeconomic inequality using SII and CI. The contribution of this study therefore lies not in being the first to assess the global self-harm burden, but in jointly examining long-term trends, fatal and non-fatal outcomes, future trajectories, and inequality patterns within a single comprehensive analysis.

### The Urgency of Addressing the Projected Increase in Self-Harm Burden

The projected increase in the global burden of self-harm by 2040, particularly regarding incidence and prevalence, underscores the urgent need for targeted prevention and intervention strategies. While there may be a slight decrease in overall DALYs, the significant rise in incidence and prevalence rates suggests that the overall impact of self-harm on population health may be stabilizing, even as the number of affected individuals is expected to grow substantially [4]. This finding supports previous research showing that non-fatal self-harm episodes represent a significant portion of the overall health burden and are strong predictors of future suicidal behavior [13]. The divergence between declining age-standardized rates and a rising absolute burden reflects the combined effects of population growth, population ageing, and persistent socioeconomic inequalities [9]. Although per capita risk has decreased in many settings, large populations in lower-SDI regions, particularly among females, remain at elevated risk. In addition, reductions in case fatality may increase the number of individuals living with the long-term consequences of self-harm. Together, these dynamics contribute to a growing global burden despite improving rates, highlighting the need for strategies that address both demographic change and structural inequities.

### Decreasing Trends in Age-Standardized Rates: A Glimmer of Hope

The observed decline in age-standardized rates of suicide-related measures from 1990 to 2021 aligns with previous studies indicating a global decrease in suicide mortality rates [4]. Notably, the more significant reduction among females compared to males underscores ongoing gender disparities in the epidemiology of self-harm [1]. Furthermore, the larger declines in high-middle SDI countries, compared to the more modest reductions in high SDI countries, suggest that socioeconomic development may influence the burden of self-harm. However, this relationship is likely complex and multifaceted [7, 8].

### Inequities in Self-Harm Burden: A Call for Targeted Interventions

The concentration index analysis shows that while inequalities in the distribution of DALYs due to self-harm have decreased over time, inequities persist, especially among females in lower-SDI populations. This finding highlights the need to address the social determinants of health and implement targeted interventions to reduce disparities in the self-harm burden [32]. Weak to moderate positive correlations between DALYs, prevalence rates, and SDI suggest that the overall burden and prevalence of self-harm tend to increase with higher levels of social development. However, significant variations between countries at similar SDI levels emphasize the complexity of self-harm behavior and the influence of factors beyond socioeconomic development [4, 21]. These persistent inequities, combined with global demographic changes, help explain why the absolute burden of self-harm is projected to increase substantially by 2040 despite declining age-standardized rates a critical consideration for resource allocation and intervention planning.

### The Importance of Sustained Prevention Efforts and Comprehensive Approaches

Long-term projections of the self-harm burden, despite significant variability, highlight the importance of ongoing efforts to prevent and mitigate the impact of self-harm worldwide. Effective prevention strategies must be evidence-based, culturally appropriate, and tailored to the specific needs of diverse populations [12, 18, 33]. Strengthening mental health systems, improving access to care, and addressing social determinants of health are essential components of a comprehensive approach to reducing the global burden of self-harm [34, 35].

### Implications for Health Policy

The results indicate a clear need for prevention strategies tailored to population risk profiles and socioeconomic contexts. In low-SDI regions, particularly among women, community-based and gender-responsive mental health programs should address violence exposure, limited autonomy, and barriers to care. In agricultural settings, reducing access to highly toxic pesticides through safer storage, substitution, and public education remains a high-impact priority. For low- and middle-income countries, improving injury surveillance, embedding mental health services within primary care, and expanding task-shifted care delivered by trained community health workers are essential to closing treatment gaps. In high-SDI countries, persistently higher mortality among men calls for targeted approaches that limit access to lethal means, encourage early help-seeking, and address work-related and social stressors. Across all regions, youth-focused school and digital interventions, alongside policies targeting poverty, gender inequality, and social isolation, are critical to mitigating the future global burden of self-harm.

### Strengths and Limitations

The primary strength of this study lies in its comprehensive global approach, leveraging the extensive GBD 2021 dataset to provide a nuanced understanding of self-harm trends across diverse populations and socioeconomic contexts. The inclusion of long-term projections offers insights for future policy and intervention planning, while analyses of socioeconomic disparities highlight areas for targeted interventions. By covering 204 countries and territories, the study ensures a global perspective on the burden of self-harm. Furthermore, the examination of multiple metrics including incidence, prevalence, mortality, YLLs, YLDs, and DALYs provides a view of the impact of self-harm, moving beyond traditional mortality-focused analyses. The incorporation of the SDI enhances our understanding of how socioeconomic factors influence self-harm trends, offering insights for policymakers and healthcare providers. However, certain limitations must be acknowledged. The accuracy of self-harm data may be compromised by underreporting and misclassification, particularly in regions with less developed health information systems. Additionally, while the GBD methodology is robust, it relies on various data sources and estimation techniques that may introduce uncertainties.

A key limitation is the heterogeneity of the GBD self-harm definition, which combines behaviors ranging from non-suicidal self-injury to completed suicide, potentially obscuring subgroup-specific patterns with distinct motivations, risk factors, and outcomes. Data quality varies substantially by socioeconomic development: underreporting is likely greatest in low-SDI settings due to incomplete vital registration, misclassification, and stigma, while high-SDI countries may still experience misclassification of ambiguous deaths and under-ascertainment of non-fatal self-harm. Middle-SDI countries show heterogeneous data quality across regions. Although GBD methods adjust for these biases, residual measurement error likely persists and should be considered when interpreting SDI-stratified estimates.

Furthermore, while our analysis includes data from 2020 to 2021, capturing the initial years of the COVID-19 pandemic, several considerations warrant attention. First, the pandemic’s impact on self-harm trends may be heterogeneous across regions and demographic groups, with some populations experiencing increased distress while others may have benefited from reduced access to means or increased social support. Second, disruptions to healthcare systems and mortality registration during the pandemic may have affected the accuracy of self-harm data in some regions. Third, the inclusion of 2020 to 2021 data in our ARIMA models may influence projections to 2040; however, the models account for this by incorporating historical patterns and UIs. Readers should interpret recent trends and future projections with these pandemic-related considerations in mind, recognizing that the full impact of COVID-19 on self-harm epidemiology may take years to fully manifest in population-level data.

The growing burden of self-harm worldwide demands urgent, targeted prevention and intervention strategies. Addressing this multifaceted health challenge requires a multifaceted approach incorporating evidence-based interventions, strengthened mental health systems, and efforts to address social determinants of health [36, 37]. Continued research and collaboration among researchers, clinicians, policymakers, and communities are essential to developing effective solutions. By harnessing innovative approaches and fostering global cooperation, we can strive to create a world better equipped to prevent and manage self-harm, ultimately promoting mental well-being and saving lives. Future efforts should focus on implementing and evaluating culturally sensitive interventions, improving data collection systems in low- and middle-income countries, and leveraging technology to extend the reach of mental health services. As the global landscape of self-harm continues to evolve, our response must be dynamic, evidence-based, and rooted in a commitment to improving mental health outcomes for all.

Our projections to 2040 carry inherent uncertainty that increases with the length forecast horizon. The wide 95% UIs reflect multiple sources of variability, including data quality heterogeneity across regions, the potential influence of pandemic-era data fluctuations (2020–2021), and the inherent unpredictability of future policy changes, socioeconomic shifts, and unforeseen global events. While central projections provide valuable guidance for policy planning, specific point estimates should be interpreted with caution.

## Conclusions

From 1990 to 2021, the global burden of self-harm showed consistent declines in age-standardized incidence, prevalence, mortality, and DALY rates, with the most pronounced improvements in high-middle SDI regions. Nevertheless, owing to population growth, population ageing, and demographic shifts, the absolute burden remains substantial and is projected to increase through 2040, particularly for incidence and prevalence. Marked sex- and age-related disparities persist, with higher mortality among males and higher non-fatal self-harm incidence among females in many regions. Although socioeconomic inequalities have narrowed over time, the burden remains disproportionately concentrated in lower socioeconomic populations, highlighting the need for sustained surveillance and targeted, equity-oriented prevention strategies.

## Availability of Data and Materials

All data in this study are available upon request by contact with the corresponding author.

## Supplementary Material

Supplementary material associated with this article can be found, in the online version, at https://doi.org/10.62641/aep.v54i3.2111.
