# Supplementary figures and images for: Bridging Three Decades: Global Self-Harm Trends From 1990–2021 and Projections to 2040
Source: Actas Esp Psiquiatr. 2026 Jun 15;54(3):644–56. doi: 10.62641/aep.v54i3.2111 (PMC13294765; doi:10.62641/aep.v54i3.2111)

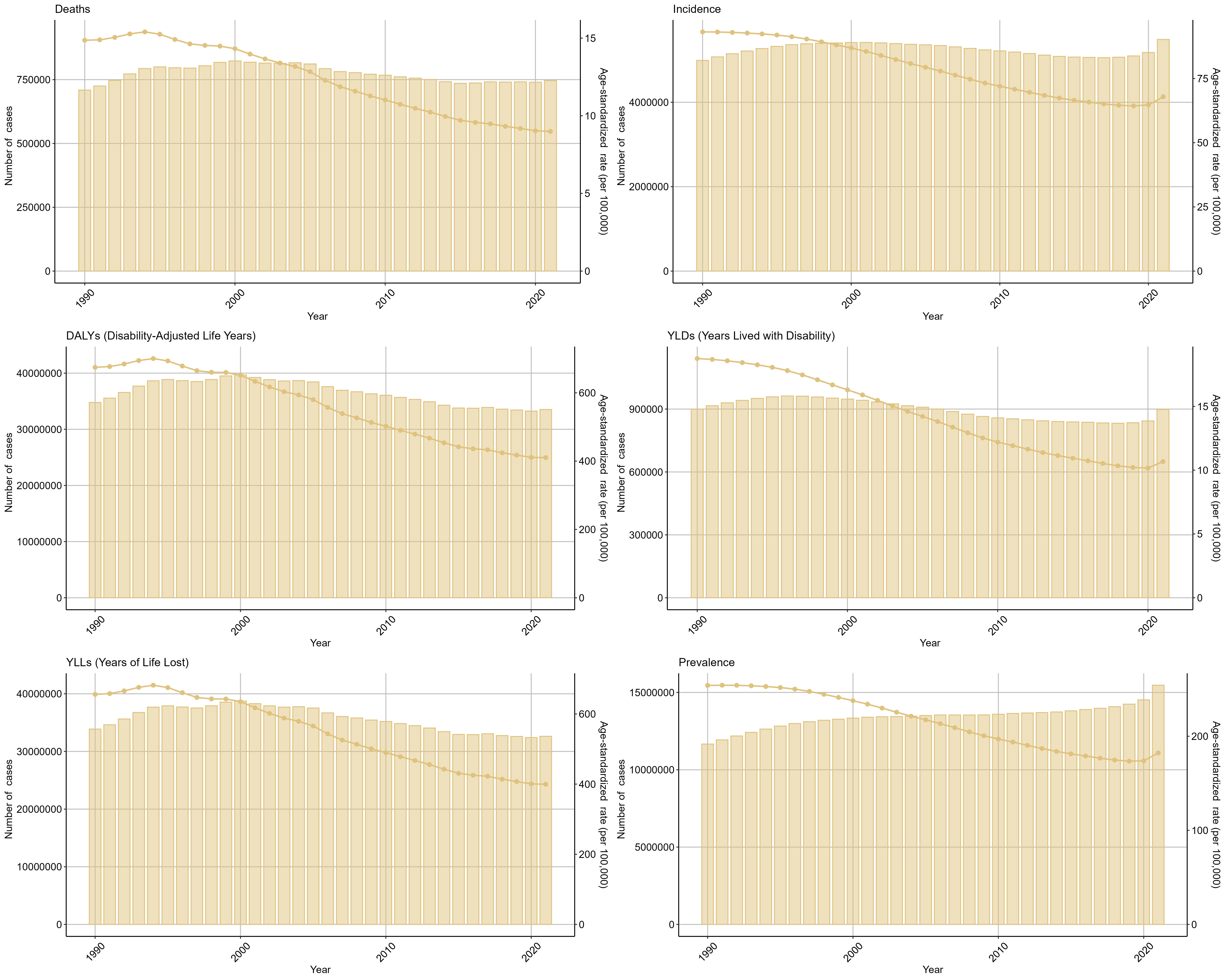

Supplement: Supplementary file 1 [file ActEsp-54-3-644-656-s1.zip › Supplementary Fig. 1.jpg]

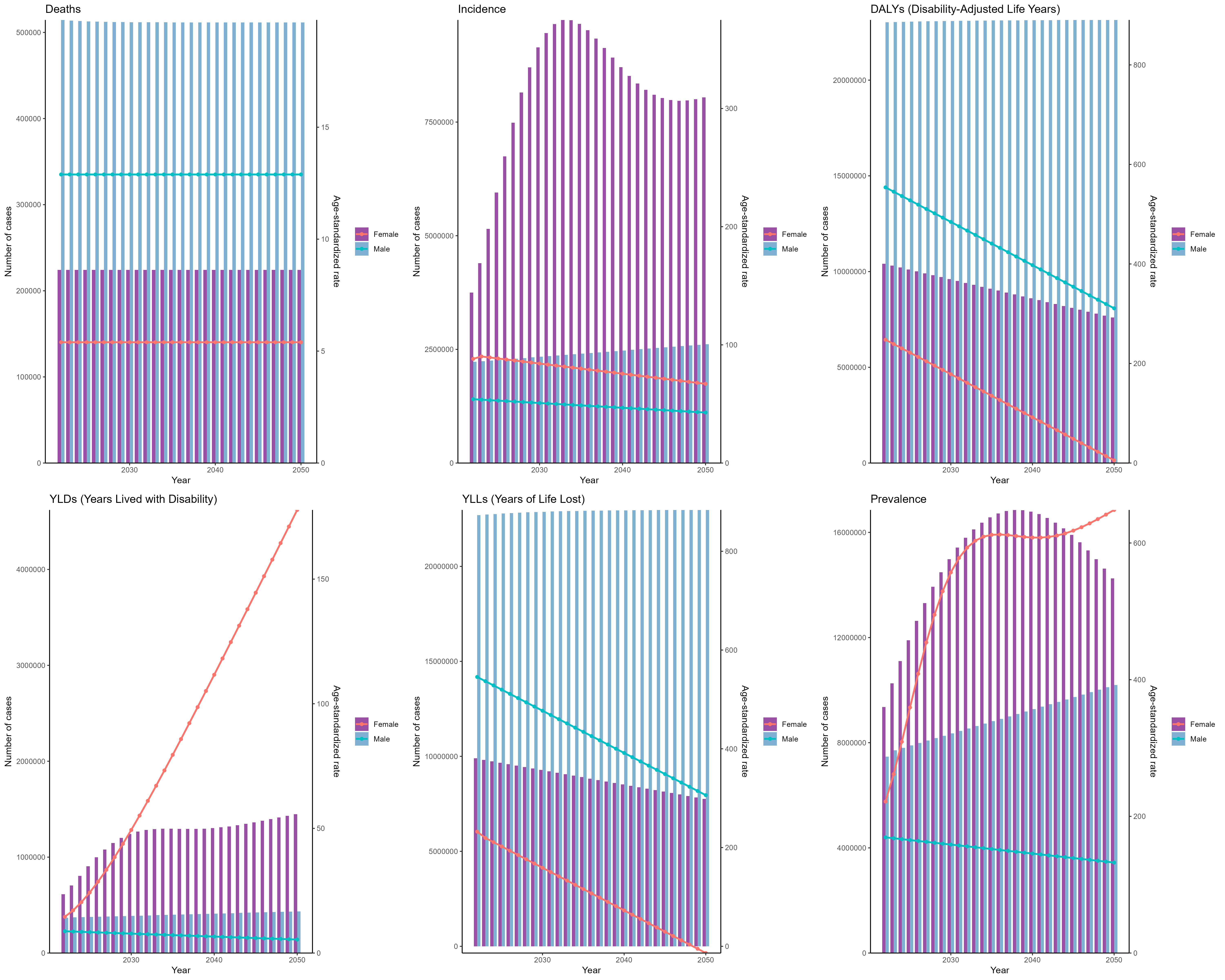

Supplement: Supplementary file 1 [file ActEsp-54-3-644-656-s1.zip › Supplementary Fig. 2.jpg]
